# Molecular alterations in hepatocellular carcinoma associated with hepatitis B and hepatitis C infections

**DOI:** 10.18632/oncotarget.7837

**Published:** 2016-03-02

**Authors:** Maria Lina Tornesello, Luigi Buonaguro, Francesco Izzo, Franco M. Buonaguro

**Affiliations:** ^1^ Molecular Biology and Viral Oncology Unit, Department of Research, Istituto Nazionale Tumori “Fondazione G. Pascale” - IRCCS, Napoli, Italy; ^2^ Hepato-Biliary Surgery Department, Istituto Nazionale Tumori “Fondazione G. Pascale” - IRCCS, Napoli, Italy

**Keywords:** hepatitis C virus (HCV), hepatitis B virus (HBV), hepatocellular carcinoma (HCC), genetic alteration, somatic mutation

## Abstract

Chronic infections with hepatitis B (HBV) and hepatitis C viruses (HCV) are the leading cause of cirrhosis and hepatocellular carcinoma (HCC) worldwide. Both viruses encode multifunctional regulatory proteins activating several oncogenic pathways, which induce accumulation of multiple genetic alterations in the infected hepatocytes. Gene mutations in HBV- and HCV-induced HCCs frequently impair the TP53, Wnt/b-catenin, RAS/RAF/MAPK kinase and AKT/mTOR pathways, which represent important anti-cancer targets. In this review, we highlight the molecular mechanisms underlying the pathogenesis of primary liver cancer, with particular emphasis on the host genetic variations identified by high-throughput technologies. In addition, we discuss the importance of genetic alterations, such as mutations in the telomerase reverse transcriptase (TERT) promoter, for the diagnosis, prognosis, and tumor stratification for development of more effective treatment approaches.

## INTRODUCTION

Liver cancer is one of the most common malignancies in the world, ranking fifth in men and ninth in women in incidence, and second among both sexes in mortality [1]. In 2012, the estimated number of new cancer cases and deaths was 782,000 and 746,000, respectively [1]. The highest incidence has been reported in Eastern and South-Eastern Asia [age-standardized rates (ASR) of 20.9 and 12.3 per 100,000 population, respectively] and Western Africa (ASR 12.1 per 100,000 population), (Table 1). On the other hand, most developed countries have low (ASR <5 per 100,000) or intermediate (ASR 5–10 per 100,000) rates with some exceptions, such as the high incidence (ASR 34.8 cases per 100,000 men) of liver cancer reported in Southern Italy [2].

**Table 1:** Estimated numbers of new liver cancer cases in males and females, crude rate and world age standardized rates [ASR(world)] per 100,000 in 2012

Population	Liver Cancer			
	Cases	Crude Rate	ASR (W)	Cumulative Risk
**Africa**	**58680**	**5.5**	**8.9**	**1.01**
Eastern Africa	7947	2.3	4.0	0.45
Southern Africa	2232	3.8	4.8	0.53
Middle Africa	5808	4.4	8.0	0.90
Northern Africa	19653	9.4	12.3	1.50
Western Africa	23040	7.2	12.1	1.30
**Asia**	**594431**	**14.0**	**13.3**	**1.46**
Eastern Asia	466336	29.4	20.9	2.26
South-Central Asia	41387	2.3	2.9	0.34
South-Eastern Asia	79953	13.2	14.2	1.64
Western Asia	6755	2.8	3.8	0.45
**Europe**	**63462**	**8.6**	**4.3**	**0.52**
Central & Eastern Europe	15953	5.4	3.1	0.37
Northern Europe	6457	6.4	3.1	0.36
Southern Europe	20558	13.1	5.9	0.71
Western Europe	20494	10.8	4.9	0.62
**Latin America & Caribbean**	**30442**	**5.0**	**4.9**	**0.57**
**Northern America**	**32718**	**9.3**	**5.8**	**0.70**
**Oceania**	**2718**	**7.2**	**5.4**	**0.60**

Ferlay J, Soerjomataram I, Ervik M, Dikshit R, Eser S, Mathers C, Rebelo M, Parkin DM, Forman D, Bray, F. GLOBOCAN 2012 v1.0, Cancer Incidence and Mortality Worldwide: IARC CancerBase No. 11 [Internet]. Lyon, France: International Agency for Research on Cancer; 2013. Available from: http://globocan.iarc.fr, accessed on 22/04/2015

Hepatocellular carcinoma (HCC) is the most common histological type accounting for approximately 70–85% of primary liver tumors [3]. Chronic HBV and HCV infections represent the major cause of HCC, being associated with more than 80% of cases worldwide [4]. Indeed, pooled estimates of lifetime relative risk to develop HCC are 15 – 20 fold higher in HBV or HCV positive patients compared to non-infected subjects [5]. Non-viral risk factors include alcoholic liver disease, non-alcoholic steatohepatitis, aflatoxin B1 dietary exposure, obesity, and diabetes [6-8]. The relative contribution of viral and non-viral factors to HCC development varies in different populations. The estimated prevalence of virus-related HCC is lower in North America (42%) and Europe (48%), and higher in Africa (80%) and Asia (87%) [4, 9]. A meta-analysis of hepatitis B surface antigen (HBsAg) and anti-HCV antibody prevalence among 27,881 HCC cases from 36 countries showed a large predominance of HBsAg in Asian, African and Latin American countries and a significant higher frequency of anti-HCV antibodies in Europe and United States [4]. The exception to these patterns is represented by the high rates of HCV-related HCC in Japan and Egypt [4].

**Figure 1:**
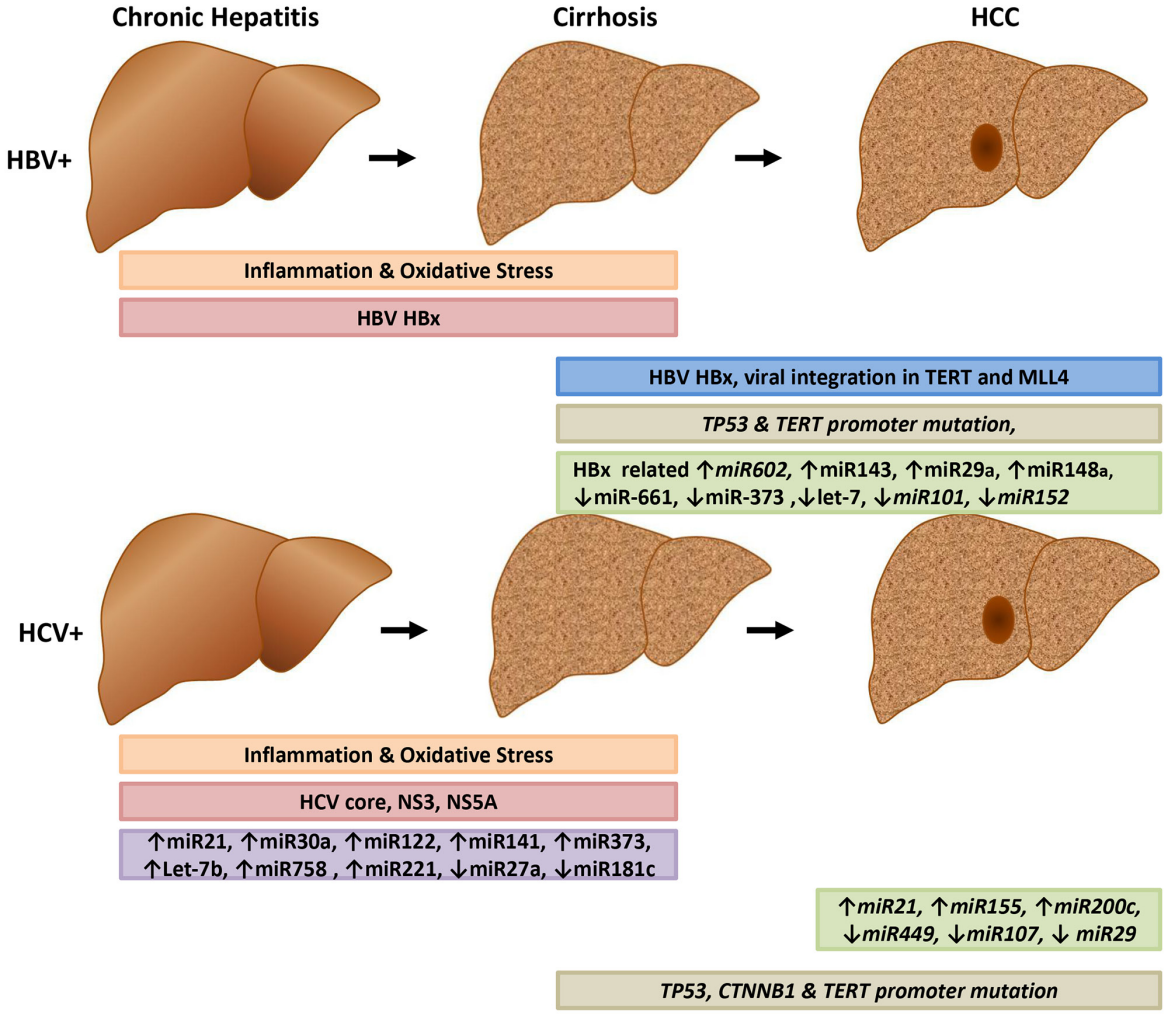
Early and late events of HBV and HCV-related liver carcinogenesis The HBV HBx protein facilitates integration of HBV into host DNA, resulting in major genetic alteration of the host genome. HBV- and HCV-encoded proteins contribute to the alteration of several signaling pathways. Both viruses promote the growth of infected cells and activate several signaling pathways including RAS, PI3K, EGFR, and IGFR1.

The HBV- and HCV-related carcinogenesis initiates in the context of chronic hepatitis, and progresses to HCC in a multistep process lasting for as long as 30 years [10] (Figure 1). During HCC progression, several environmental factors (aflatoxin B1, alcohol consumption, cigarette smoking, hepatotoxic chemical agents) as well as host co-factors (elevated serum androgen levels, genetic polymorphisms, DNA repair enzymes) may synergize and lead to progressive accumulation of multiple genomic changes in the hepatocytes [11, 12]. Among these, non-synonymous mutations in *TP53* and *CTNNB1* genes are well known cancer drivers for HCC development with variable frequencies depending on the underlying etiology [13, 14].

Over the last decade, massively parallel sequencing technologies allowed to further uncover the genomic diversity of HCC and to identify consistent gene alterations activating signaling pathways relevant to cell transformation [15, 16]. Such analyses allowed to identify HCC subgroups characterized by definite genetic profiles that may be linked to specific oncogenic factors and are useful to further stratify HCCs for personalized medicine applications [17].

Here, we review the molecular pathogenesis of primary liver cancer with particular emphasis on the host genetic variations identified by high-throughput technologies in the context of HBV and HCV related HCC. We discuss the importance of genetic alterations in diagnosis, prognosis as well as in tumor stratification for more efficient treatment approaches.

### HBV and hepatocellular carcinoma

HBV is a partially double-stranded hepatotropic DNA virus containing four partial overlapping open reading frames (ORFs) encoding the reverse transcriptase/polymerase (Pol), the capsid protein (core antigen HBcAg), three envelope proteins (L, M, and S) and the transactivating protein x [18].

HBV infection contributes to hepatocarcinogenesis by different mechanisms including 1) expression of HBx protein; 2) integration of viral DNA into the host genome; and 3) accumulation of somatic mutations in human genes with or without exposure to other carcinogens (i.e. aflatoxin B1), [10, 19, 20].

### HBV HBx protein

The HBV protein HBx transactivates viral and cellular genes by interacting with nuclear transcription factors, such as cyclic adenosine monophosphate(cAMP) response element-binding protein (CREB), activating protein 1 (AP-1), nuclear factor kappa B (NF-kB), and specificity protein 1 (Sp-1). HBx affects also several cellular pathways including DNA repair, cell proliferation, differentiation and apoptosis [20-24]. In addition, HBx protein trans-activates DNA methyltransferase 1 (DNMT1) and DNMT3A genes in the HBV infected hepatocytes, resulting in the suppression of cell cycle regulators P16INK4A and p21 Cip1/CDKN1A, cell-adhesion molecule E-cadherin as well as SFRP1 and SFRP5 genes, which inhibit Wnt signaling pathway [25-30]. Moreover, Wnt/β-catenin pathway is directly activated by HBx protein, which interferes with proteasomal degradation of β-catenin [31, 32]. More recently, HBx has been shown to activate the Yes-associated protein (YAP) oncogene, a downstream effector of the Hippo-signaling pathway, which represents a key element in HCC development [33]. The HBx protein can also bind to the p53 oncosuppressor, leading to the disruption of the p53/XPB/XPD complex of the transcriptional factor II H and compromising the nucleotide excision repair mechanism [34]. Recent studies showed that HBx is able to activate AKT, favoring persistent, non-cytopathic HBV replication and inhibition of the transcription factor hepatocyte nuclear factor 4 (HNF4) [35].

### HBV integration and chromosomal alterations

HBV genome commonly integrates in HCC causing global genomic instability, increased expression of genes adjacent to integration loci, and expression of viral-host fusion transcripts [36-39]. Genome-wide analysis showed that HBV integration occurs in 86% of HCCs and in 30.7% of adjacent non-tumor tissues [40]. A similar frequency (75.5%) has been identified in HCC patients with occult HBV infection [41]. The analysis of genome instability showed that somatic copy number variations are significantly increased at locations adjacent to HBV integration sites [40], and that the number of chromosomal aberrations correlates with the mutational status of tumor suppressor genes, such as TP53, RB1, CDNK2A and TP73 [42]. Next-generation sequencing uncovered several new genes recurrently interrupted by HBV integrants including TERT, MLL4, CCNE1, NTRK2, IRAK2 and p42MAPK1 [40, 43-45]. The integration of HBV DNA preferentially involves the HBx sequence which frequently undergoes deletion at the 3′-end, causing the expression of a C-terminal-truncated HBx protein able to enhance HCC cell invasiveness and metastasis [46, 47]. Transcription profiling by RNA-sequencing analysis allowed identification of several viral-human fusion transcripts generated as a consequence of HBV integration. The most abundant is the long non-coding RNA HBx-LINE1 chimera, which has been detected in 23% of HBV-related HCCs [42], and has been shown to promote tumor growth through the activation of Wnt/b-catenin signaling [48].

### HBV and aflatoxin B1 interaction

In HBV-associated HCC, there is a strong overrepresentation of TP53 mutations, particularly in geographic regions endemic for HBV and with dietary exposure to aflatoxin B1 (AFB1) [49]. Specifically, AFB1 induces a non-synonymous mutation (G to T transversion) changing arginine to serine at codon 249 of TP53 gene in up to 50% of HCCs. The mutated p53, together with chronic HBV infection, synergistically increase the risk to develop HCC [49, 50]. Indeed, the p53 R249S is able to bind the HBx protein and to promote hepatocyte transformation [51].

### HCV and hepatocellular carcinoma

HCV is a single-stranded RNA virus encoding a large polyprotein of 3,000 amino acids. The HCV polyprotein can be cleaved by viral and cellular proteases into four structural proteins (capsid protein C, envelope glycoproteins E1 and E2, and protein P7), and six non-structural proteins (NS2, NS3, NS4A, NS4B, NS5A, and NS5B) [52].

HCV causes chronic hepatitis in more than 80% of infected subjects, versus the 10% in HBV infected patients, and is up to 20 fold more efficient than HBV in promoting liver cirrhosis [53]. Pathogenesis of HCV-related HCC mainly relies on the ability of the virus to cause chronic inflammation, immune-mediated hepatocyte death, tissue damage, fibrosis and evolution to cirrhosis [54-56]. The HCV core protein C as well as the non-structural proteins NS3, NS5A, and NS5B induce hepatocarcinogenesis through their ability to perturb several cellular pathways, such as DNA repair, proliferation and apoptosis [57-59].

### HCV core protein

The HCV core protein binds to numerous transcription factors, thus regulating expression of several host genes [60, 61]. In addition, it promotes cell growth and survival by activation of mitogen-activated protein kinase (MAPK) signaling cascade, including MEK1, ERK1/2, JNK, p38 MAP kinases, and MKP1 Map kinases [62-64]. HCV core enhances cell proliferation by inhibiting the synthesis of p53, p21 CDK inhibitor, and E2F-1 as well as the phosphorylation of pRb [65]. Moreover, it is able to suppress immune-mediated apoptosis by inhibiting caspase-8 via over-expression of the cellular FADD-like interleukin-1 converting enzyme (c-FLIP) [66]. In addition, it enhances angiogenesis by triggering the production of TGF-β2 and VEGF proteins, and stabilizing the hypoxia-inducible factor 1 (HIF-1a) [67]. HCV core protein induces IL-6, gp130, leptin receptor, and STAT3 over expression, which in turn may deregulate c-Myc and cyclin D1 downstream the STAT3 signaling pathway [68]. HCV core protein also activates the Wnt/b-catenin cascade, which is known to play a significant role in the HCC development [69].

### HCV NS3 protein

The HCV NS3 protein is a multifunctional protein with protease, RNA helicase, and NTPase activity. NS3 can promote hepatocarcinogenesis by its binding with certain cellular proteins, such as p21 and p53 [70]. Recently, HCV NS3/4A protease was demonstrated to activate the EGFR signaling pathway through the proteolytic cleavage of tyrosine phosphatase T-cell protein (TC-PTP), resulting in increased EGFR activity and the downstream PI3K/Akt pathway [71]. MAP kinase signaling, through activation of JNK, was also implicated in HCV NS3 protein-mediated cell growth in infected cells [65].

### HCV NS5A protein

NS5A has been shown to bind a wide range of cellular proteins controlling signal transduction and host microenvironment [72]. Particularly, the truncated HCV NS5A protein localizes to the nucleus and acts as a transcriptional activator. NS5A can bind cellular signaling components and regulatory protein kinases, leading to the suppression of the host immune response and inhibition of apoptosis [73]. NS5A binds and stabilizes β-catenin, inducing activation of the c-Myc promoter and increased c-Myc expression, which increases production of reactive oxygen species, DNA damage, and cell-cycle deregulation [74, 75]. NS5A also stabilizes poly(ADPribose) polymerase 1 (PARP-1), which is involved in DNA repair and apoptosis, thus contributing to genetic instability and accumulation of mutations in HCV-infected hepatocytes [59, 76, 77].

### Gene expression profiling in HCC

Early studies on gene expression profiling highlighted the wide heterogeneity of global gene expression patterns in liver tumors [78, 79]. Hierarchical clustering analysis of tumor-specific genes contributed to classify HCC subtypes, unravel the complex pathogenesis of HCC and stratify tumors according to their etiological factor, clinical stage, recurrence rate, and prognosis [80-82]. Several reports showed strong expression signatures in genes regulating cell proliferation and anti-apoptotic pathways (i.e., PNCA and cell cycle regulators CDK4, CCNB1, CCNA2, and CKS2), ubiquitination mechanisms [83, 84], as well as molecular markers of tumor progression like HSP70, CAP2, GPC3, and GS [85]. A class-comparison analysis performed in our lab (HCV-related HCC, HCV-related non HCC and metastatic liver tissue vs. normal control; HCV-related HCC vs. autologous HCV-related non HCC liver tissue) identified a gene-set that distinguish the different types of liver disease [86]. In particular, the time course analysis allowed to identify several candidate genes as progression markers (e.g., GPC3, CXCL12, SPINK1, GLUL, UBD, TM4SF5, DPT, SCD, MAL2, TRIM55, COL4A2) [86]. Altogether, these data are useful for developing a specific gene-chip including those genes showing the highest fold increase.

Moreover, HCC-specific alterations of signal transduction pathways and protein expression patterns have been detected and opened opportunities for new therapies targeting molecular factors such as EGFR, VEGF, DDEFL, VANGL1, WDRPUH, ephrin-A1, GPC3, number gain 7q, PFTK1, PEG10 and miR-122a [87, 88].

### miRNA in HBV-related HCC

Several microRNAs (miRNA) have been found deregulated in HBV-positive HCCs. The HBx protein has a major role in the miRNAs alteration. Specifically, HBx is able to increase the expression of miRNA 602, targeting the putative tumor suppressor Ras association domain family 1 isoform A (RASSF1A) [89]; miRNA-143, targeting fibronectin type III domain-containing 3B (FNDC3B) promoting hepatoma cell invasion, migration and tumor metastasis [90]; miRNA-29a and miR-148a, targeting phosphatase and tensin homolog (PTEN) and stimulating cell migration [91, 92]. Notably, levels of miR-122 and let-7b have been found increased in the serum of HBV-positive patients with early HCC and have been proposed as useful markers to differentiate early HCC from dysplastic nodules [93]. In addition, HBx inhibits the expression of miR-661, targeting metastasis associated 1 factor (MTA1) [94]; miR-373, targeting cadherin 1 (CDH1) gene [95]; let-7a, which is implicated in the cell proliferation control through STAT3 modulation [96]; and miR-101 and miR-152, controlling the expression of DNMT3A and DNMT1, respectively [97, 98]. HBx also inhibits expression of miR-148a, which targets hematopoietic pre-B cell leukemia transcription factor interacting protein (HPIP), and miRNA-16 family, targeting cyclin D1 (CCND1); both mi-148a and miRNA-16 family are associated with tumor growth control [99, 100].

### miRNA in HCV-related HCC

HCV replication and pathogenesis are tightly controlled by the expression of several miRNAs [101]. In particular, miRNA-122 favors HCV replication by binding directly to viral RNA, while miRNAs-130a and-21 subvert the IFN signaling pathway, leading to immune evasion [101, 102]. miRNAs-196/199a and -448/let-7b attenuate viral replication, and Let-7b and miRNA-221 compromise the antiviral effect of IFN-α [103, 104]. Expression of miRNAs regulating lipid metabolism (miRNA-27a) and hepatocyte growth (miRNA 181c) is decreased in HCV infected cells. In contrast, expression of miRNA-155, promoting hepatocyte proliferation and inflammation, and miRNA-21 and -200c, promoting fibrosis, is increased in advanced stages of liver disease [101].

### Chromosomal aberrations and gene copy number variations in HCC

Chromosomal alterations are very common in liver tumors. Comparative genomic hybridization (CGH) data showed frequent gain of chromosomal regions 1q (57.1%), 8q (46.6%), 6p (22.3%), and 17q (22.2%), and prominent losses of 8p (38%), 16q (35.9%), 4q (34.3%), 17p (32.1%), and 13q (26.2%), Table 2, [105, 106]. Chromosome losses in the regions 4q, 13q, 16q, and 8p are more frequent in HBV - related tumors, while loss of chromosome 8p in HCV-positive HCCs is less frequent compared to virus negative tumors [105]. Moreover, gains of 1q and 8q as well as losses of 4q, 16q and 13q have been shown to increase with HCC progression [105].

**Table 2:** Major chromosomal alterations (frequency above 20%) identified in 31 studies by conventional metaphase-based CGH analysis

Chromosome Gain	Hot spot	All HCC (n=785)	HBV HCC (n=244)	HCV HCC (n=110)
1q	1q31	57.1%	53.3%	45.5%
6p	6p25–p23	22.3%	24.2%	16.4%
8q	8q24.2	46.6%	46.7%	34.5%
17q	17q25	22.2%	20.9%	17.3%
**Chromosome Loss**				
4q	4q23–24	34.3%	43.4%	27.3%
8p	8p21.3–p21.2	38.0%	40.6%	20.0%
13q	13q21.1–q21.3	26.2%	31.1%	23.6%
16q	16p13.2	35.9%	41.8%	27.3%
17p	17p12	32.1%	32.4%	30.9%

High-resolution array CGH studies allowed to discover chromosomal gains in 5p15.33 and 9q34.2–34.3 and losses in 6q, 9p and 14q, in addition to the previously identified genetic aberrations [107-111]. Copy number variation of 1q21.3-44 and LOH of 1p36.21-36.32 and 17p13.1-13.3 regions were identified in early HCC but not in chronic liver disease, suggesting their possible causative role in HCC development, while gains of 5q11.1-35.3, 6p, and 8q11.1-24.3 as well as LOH of 4q11-34.3 and 8p11.21-23.3 appear associated with more advanced tumor stages [108, 112]. The copy number gain of 8q24 region is generally associated with an increased expression of c-Myc gene, particularly in viral and alcohol-related HCCs but not in cryptogenic HCCs [111]. Other small chromosomal aberrations, such as amplification of 1q32.1 and 20q13.33, have been associated with overexpression of MDM4 and EEF1A2, respectively, in approximately 50% of tumors, independently from the etiology [111]. The integration of CGH data with gene expression arrays allowed to identify over-expressed candidate oncogenes, such as TAGLN2, MDM4, SNRPE, SPP1 VEGFA, PEG10, Jab1, HEY1, BOP1 and EEF1A2 [109, 111, 113-117] and down-regulated candidate tumor suppressor genes, such as TRIM35, DLC1, CRYL1, and Spry2 [117-120]. Few studies analyzed the prognostic significance of chromosomal alterations and gene profile expression [121]. Roessler et al. combined CGH data and gene expression arrays of 256 HCC cases, and identified 10 genes associated with poor survival, of which six were located at chromosome 8p, [122].

### Somatic mutations in HBV and HCV-related HCC

Genomic instability of viral-related HCCs is characterized by high frequency of somatic mutations. Several studies showed that TP53 oncosuppressor and CTNNB1 oncogene are the most frequently mutated genes in primary liver cancer, being identified in about 25% and 30% of HCCs, respectively [13]. Up to 75% of missense TP53 mutations, other than the R249S induced by AFB1, are scattered over 200 codons of the TP53 region encoding for the DNA-binding domain [123-126], and show similar frequencies in HCCs with different etiologies [13]. Such a finding suggests that chronic inflammation, reactive oxygen species, and oxidative DNA damage, which are common effects of cancer causing factors, may be responsible for such variations. TP53 mutations may cause several pathway deregulations in HCCs. Okada et al. identified 83 genes differentially expressed in TP53 mutant compared to wild type TP53 liver tumors [127]. The genes differentially expressed in TP53 mutant tumors include cell cycle regulators (CCNG2, BZAP45) and cell proliferation-related genes (SSR1, ANXA2, S100A10, and PTMA) [127]. These data support the hypothesis that p53 mutant tumors have higher malignant potentials compared with wild type p53 [128, 129].

CTNNB1 gene, expressing β-catenin, and AXIN1 and AXIN2 genes, encoding for components of β-catenin degradation complex, are frequently mutated in liver cancers [130, 131, 131-133]. Interestingly, CTNNB1 mutations have been shown to occur mainly in alcohol and HCV-related tumors [16, 134, 135]. Guichard et al. reported CTNNB1 mutations in 11.4% and 33.3% of HBV and HCV-related HCCs, respectively, and in 41.8% of alcohol-related HCCs [16]. In addition, mutations in CTNNB1 and TP53 genes appear to be mutually exclusive, suggesting that inactivation of either pathway is sufficient to induce cell transformation [16].

Next generation sequencing allowed to identify other oncosuppressor genes in HCC, which have lower mutation frequencies compared to TP53, independently from the etiology of the tumor. They include P16INK4 (6%–17%), P14arf (5%), AXIN1 (5%–15%), AXIN2 (2%–10%), TIP30 (24%), IGFR2 (10%–20%), KLF6 (15%), Caspase- 8 (13%), PTEN (5%–8%), etc. [14, 135-137]. Moreover, oncogenes other than CTNNB1 are less frequently mutated and they include EGFR (1%) and Erb2 (2%), K-ras (0%–19%) and N-ras (2%) and PIK3CA (<5%) [14, 135-137].

At least three large whole-exome sequencing studies described the mutational landscape and possible druggable targets in viral-related and viral-unrelated HCC, Table 3, [138-140]. Totoki et al. identified 30 candidate driver genes associated with 11 core pathways in 608 liver cancers including 413 cases from Japan [138]. Importantly, they discovered that 68% of HCC cases had telomerase reverse transcriptase (TERT) genetic alterations, including promoter mutation, focal amplification, and viral genome integration, and recognized TERT as a central regulator of hepatocarcinogenesis.

**Table 3:** Comparison of recurrently mutated genes in HCC identified in three large studies in Japan, Korea and Europe

Function	Gene Name	Totoki et al.* (n=452)	Ahn et al.* (n=231)	Schulze et al.* (n=243)
WNT/β-catenina	CTNNB1	31.0%	22.9%	37.4%
	RSPO2	-	3.0%	-
	AXIN1	6.2%	6.9%	11.1%
	FZD6	-	3.0%	-
Chromatin remodeling	ARID1A	8.6%	3.9%	12.8%
	ARID2	10.8%	3.0%	6.8%
	ARID4b	1.1%	3.0%	-
p53/cell cycle	TP53	32.2%	31.2%	24.3%
	CDKN2A	2.2%	6.1%	8.5%
	ATM	4.4%	2.2%	5.5%
	CDKN2B	0.2%	2.2%	5.1%
	CCND1	-	5.2%	4.7%
	RB1	4.2%	7.8%	3.8%
	RBL2	2.2%	3.0%	-
	HUWE1	0.4%	0.9%	3.4%
Epigenetic regulation	MLL2	3.8%	5.2%	5.5%
	MLL3	2.4%	3.0%	2.0%
	MLL	2.9%	3.9%	-
	CHD1	-	3.0%	-
	CHD7	0.9%	3.0%	3.4%
	CREBBP	2.0%	3.0%	3.0%
	SMC3	-	-	3.0%
	SRCAP	2.9%	-	3.0%
Telomere mantainence	TERT promoter	55.1%	-	60.0%
	TERT	4.0%	-	-
Oxidative stress	NFE2L2	4.9%	3.0%	6.4%
	KEAP1	2.4%	-	3.8%
Hepatic differentiation	ALB	6.9%	4.8%	12.8%
	APOB	10.2%	10.3%	9.4%
	HNF1A	2.2%	0.9%	4.7%
	FGA	1.5%	1.7%	3.4%
MAPK	RPS6KA3	3.8%	5.0%	6.8%
	FGF4	0.2%	-	4.7%
	FGF19	-	5.0%	4.7%
	FGF3	0.2%	0.9%	4.3%
	EPHA4	1.8%	2.6%	3.4%
	FLT4	0.9%	2.6%	3.4%
	HGF	0.7%	0.4%	3.0%
	NTRK3	1.5%	1.7%	3.0%
PI3K-AKT-mTOR	TSC2	5.3%	3.0%	5.1%
	FGF4	0.2%	-	4.7%
	FGF19	4.0%	5.0%	4.7%
	FGF3	0.2%	0.9%	4.3%
	FLT4	0.9%	2.6%	3.4%
	PTEN	1.3%	2.0%	3.4%
	HGF	0.7%	0.4%	3.0%
	PRKCB	1.5%	3.5%	-
	NTRK3	1.5%	1.7%	3.0%
	JAK3	0.9%	0.4%	3.0%

* Totoki et al. (n=413 Japan, 92 HBV+ and 183 HCV+); Ahn et al. (n=231 Korea, 167 HBV+ and 22 HCV+); Schulze et al. (n=243 [n=193 France, n=41 Italy, n=9 Spain], 33 HBV+ and 61 HCV+).

Ahn et al. analyzed 231 cancer cases from Korea and identified recurrent somatic mutations in nine genes, comprising TP53, CTNNB1, AXIN1, RPS6KA3, and RB1, homozygous deletions in FAM123A, RB1, and CDKN2A, and high-copy amplifications in MYC, RSPO2, CCND1, and FGF19 [139]. RB1 mutations were associated with cancer recurrence in resectable HCCs. Schulze and colleagues identified 161 putative driver genes associated with 11 pathways: TERT expression, WNT/β-catenin, PI3K-AKT-mTOR, TP53 – related pathway, MAP kinases, hepatic differentiation, epigenetic regulation, chromatin remodeling, oxidative stress, IL-6/JAK-STAT and TGF-β [139, 140]. Approximately 30% of liver tumors analyzed in these studies harbored genetic alterations potentially targetable by Food and Drug Administration (FDA)-approved drugs [140]. The analysis of copy number variations revealed recurrent homozygous deletions of the CFH locus, IRF2, CDKN2A, PTPN3, PTEN, AXIN1 and RPS6KA3 and recurrent focal amplifications of TERT, VEGFA, MET, MYC, the FGF-CCND1 locus containing FG3, FG4 and FGF19, JAK3 and CCNE1 [140]. Moreover, integrating results of exome sequencing mutation and focal copy number alteration allowed to identify 3 groups of putative cancer driver genes: CTNNB1, TP53 and AXIN1 clusters [140].

The frequency of mutations in different genes seems related to the cancer etiology. TP53 gene was mostly mutated in HBV-related HCC, while CTNNB1, TERT, CDKN2A, SMARCA2, and HGF genes were mainly mutated in alcohol-related HCCs, and IL6ST was mutated in HCCs with no known etiology. Conversely, no specific gene mutation was associated with HCV infection, metabolic syndrome and hemochromatosis [138-140].

Somatic mutations in the TERT promoter have been identified as the first recurrent genetic alteration in 25% of dysplastic cirrhotic nodules [141, 142]. These mutations create a consensus binding sequence for a ternary complex factor and induce expression of telomerase reverse transcriptase [148]. Conversely, several other genes known to be recurrently mutated in HCC, including CTNNB1, TP53, ARID1A, ARID2, RPS6KA3, NFE2L2 and KEAP1 were not mutated in dysplastic nodules [141]. TERT promoter mutations may be considered as biomarkers for the identification of premalignant lesions developed in cirrhosis patients with a high risk of progression to HCC.

### Immunotherapeutic approaches and gene mutations in HCC

In several cancer types, the activity of tumor antigen-specific T-cells is tightly regulated by the balanced expression of stimulatory and inhibitory molecules defined as “immune checkpoints” [144, 145]. Therapies targeting these checkpoints, such as those directed against cytotoxic T-lymphocyte antigen 4 (CTLA-4) and programmed death 1 receptor (PD-1), have shown to be more effective in cancers characterized by high rates of somatic mutations [146]. Recent studies have indicated that a high tumor mutation burden increases responsiveness to CTLA-4 inhibition in melanoma, to PD-1 inhibition in non-small cell lung cancer and in mismatch repair-deficient colorectal cancers [147]. The hypothesis is that the higher number of genetic variations leads to a greater number of mutated epitopes in tumor proteins (neoantigens). Such neoantigens may be characterized by an improved MHC-binding profile, resulting in superior presentation to T cells for eliciting a stronger cytotoxic response [148, 149]. Very recently, this has been experimentally proven in animal models [150, 151] as well as in melanoma patients treated with the anti–CTLA-4 monoclonal antibody, ipilimumab [152, 153].

Liver sinusoidal endothelial cells express high levels of the inhibitory molecule program death receptor ligand 1 (PD-L1) and low levels of the co-stimulatory molecules CD80 and CD86, thereby limiting their ability to effectively activate CD4-positive (CD41) and CD8 1 T lymphocytes [154, 155]. Immune checkpoint inhibitors have been recently evaluated in HCC patients. The anti-CTLA-4 monoclonal antibody tremelimumab showed a safe profile and antitumor activity in HCC patients with chronic HCV infection [156]. Very recently, results from a phase I/II clinical trial (ClinicalTrials.gov Identifier: NCT01658878) presented at the last 2015 ASCO Meeting showed that nivolumab, a fully humanized IgG4 monoclonal antibody to PD-1, may be a promising treatment for patients with advanced HCC [157]. Indeed, the overall survival at 1 year was 62% and overall objective responses rate was 19%, including complete response (CR) in 5% and partial response in 14% of enrolled patients. Such responses are significantly higher compared to responses to the kinase inhibitor sorafenib, the current standard of care for late stage HCC.

Further studies are needed to evaluate whether the immune responses elicited by mutated epitopes could lead to an increased efficacy of anti immune-checkpoint therapies also in liver cancer.

## CONCLUSIONS

Classification of liver cancers in homogeneous sub-groups characterized by specific molecular alterations is an important tool for the application of personalized therapies. Several commonly altered pathways have emerged following the integration of data obtained with multiple high-throughput analyses. Common oncogenic drivers, differentially represented in HCCs with different etiologies, include genetic alterations affecting TERT, Wnt/beta-catenin, JAK/STAT and PI3K-AKT-mTOR pathways. Drugs targeting these pathways are now available and have been approved in clinical trials.
